# Community Point of Care Testing in Diagnosing and Managing Chronic Kidney Disease

**DOI:** 10.3390/diagnostics14141542

**Published:** 2024-07-17

**Authors:** Rouvick Mariano Gama, Danilo Nebres, Kate Bramham

**Affiliations:** 1 Department of Inflammation Biology, Faculty of Life Sciences and Medicine, School of Immunology and Microbial Sciences, Sir James Black Centre, King’s College London, 125 Coldharbour Lane, London SE5 9NU, UK; 2 King’s Kidney Care, King’s College Hospital, Denmark Hill, London SE5 9RS, UK; 3 Department of Women and Children’s Health, School of Life Course and Population Sciences, Faculty of Life Sciences and Medicine, King’s College London, London SE5 9RJ, UK; 4 Centre for Nephrology, Urology and Transplantation, School of Immunology and Microbial Science, Faculty of Life Sciences and Medicine, King’s College London, London SE5 9RJ, UK

**Keywords:** point-of-care, creatinine, estimated glomerular filtration rate (eGFR), chronic kidney disease (CKD)

## Abstract

Chronic kidney disease (CKD) poses a significant global health challenge with increasing prevalence and associated morbidity. Point-of-care testing (POCT) provides an opportunity to improve CKD management and outcomes through early detection and targeted interventions, particularly in underserved communities. This review evaluates the roles of POCT in CKD, focusing on utility (through screening programs, monitoring of kidney function, and assessing participants on renally excreted medications), accuracy, and acceptability. Screening programs employing POCT have demonstrated promising outcomes, with improved rates of CKD diagnosis in groups with disparate health outcomes, offering a vital avenue for early intervention in high-risk populations. These have been conducted in rural and urban community or pharmacy settings, highlighting convenience and accessibility as important facilitators for participants. In addition, POCT holds significant promise in the monitoring of CKD, particularly in groups requiring frequent testing, such as kidney transplant recipients and patients on renin-angiotensin-aldosterone inhibitors. The consideration of the variable analytical performance of different devices remains crucial in assessing the utility of a POCT intervention for CKD. While the convenience and improved accessibility of home self-testing versus healthcare professional management is important, it must be balanced with acceptable levels of accuracy and precision to maintain patient and clinical confidence. Despite challenges including variability in accuracy and the user-friendliness of devices, patient feedback has generally remained positive, with studies reporting increased patient satisfaction and engagement. However, challenges regarding wider uptake are limited by healthcare professional confidence (in test reliability), the potential for increased workload, and early prohibitive costs. In conclusion, POCT represents a growing and valuable tool in enhancing CKD care, particularly in resource-limited settings, but careful consideration of device selection and implementation strategies is essential to achieve desired outcomes.

## 1. Introduction

Chronic kidney disease (CKD) is a growing global public health issue that currently affects between 9 and 13% of the population worldwide. In combination with acute kidney injury (AKI), it is predicted to be a top-five cause of mortality by 2040 [1,2,3]. The prevalence, incidence, and progression of kidney disease varies between and within countries, influenced by access to healthcare, political systems, available technology, and socioeconomic status, with people in the lowest socioeconomic quartile having up to 60% increased risk of progressive CKD compared to those in the highest quartile [1,4,5,6].

The most common causes of CKD worldwide are diabetes mellitus, accounting for 30–50% of people with CKD (80% in low- and middle-income countries) and hypertension [7,8], both of which are also disproportionately overrepresented in young ethnic minority populations in high-income countries (e.g., in Black and South Asian populations in the United Kingdom), and are associated with an increased risk of accelerated decline in kidney function [9,10,11].

Diagnosis of CKD is made using estimated glomerular filtration rate (eGFR), calculated primarily using isotope dilution mass spectrometry (IDMS)-traceable creatinine, age, biological sex, and the urinary albumin: creatinine ratio (uACR) [12]. An alternative or adjunct to creatinine is cystatin-C. This has been shown to improve eGFR estimation, particularly when used in conjunction with creatinine. It is therefore recommended by the KDIGO CKD 2023 guidelines for confirmatory CKD testing and is utilised in paediatrics and parts of mainland Europe [12]. However, it has still not been widely adopted (e.g., it is not recommended by the National Institute for Health and Care Excellence in the UK, due to the risk of false positives) and is not readily available in a number of laboratories [13,14]. Furthermore, there are no studies to our knowledge using POCT for cystatin-C; therefore, it will not be discussed in this review.

CKD is associated with an increased risk of end-stage renal failure (ESRF), cardiovascular events, and premature death. A health economic analysis in the UK published in 2011 reported that CKD was responsible for an excess of 40,000 premature deaths, 12,000 excess myocardial infarctions, and 7000 excess cerebrovascular accidents each year in the United Kingdom alone [15].

As CKD is irreversible and progressive, organisations such as the National Institute for Health and Care Excellence (NICE) in the United Kingdom have placed emphasis on the early detection of the disease to facilitate intervention and slow the progression of the disease and its associated comorbidities [13].

Rates, detection, and management of kidney disease tend to be better defined in high-income countries; however, emerging evidence suggests that low- and middle-income countries are likely to have similar if not greater burdens of disease [16,17]. Furthermore, detection may be limited by healthcare infrastructures such as proximity to routine testing, costs, and available workforce.

Point-of-care testing (POCT), also referred to as ‘rapid tests’, is defined as in vitro procedures that are performed in close proximity to patients and are characterised by a rapid turnaround time [18,19]. Aligned with growing expectations for timely investigations and management amongst patients, the market for POCT has been rapidly increasing. It was worth approximately USD 44 billion in 2022 and is expected to rise to USD 78 billion by 2030 [20]. Within this market, kidney function testing has also been advancing with a range of POCT devices available for most routine renal biochemistry tests and urinalysis.

The purpose of this review is as follows: (1) summarise the available devices, with a focus on accuracy and precision; (2) discuss the roles of POCT in CKD; and (3) describe current healthcare professional and patient/service user perspectives as a potential facilitator or barrier to the wider use of POCT in daily clinical practice.

## 2. POCT Devices for Kidney Disease: Accuracy, Precision and Acceptability

The diagnosis and classification of CKD is based on the estimated glomerular filtration rate (eGFR) and urinary albumin: creatinine ratio (UACR), as defined by the Kidney Diseases: Improving Global Outcomes (KDIGO) CKD 2023 Guidelines [12]. eGFR is calculated using an equation derived from age, biological sex, and endogenous filtration marker concentration, of which creatinine, a waste product from predominantly skeletal muscle, is most commonly used worldwide. It is measured using either the modified Jaffe or enzymatic assays with isotope dilution mass spectrometry (IDMS) as a reference standard and quantified in units of mg/dL or μmol/L. Turnaround time for the assay is usually a few hours; however, accounting for sample delivery and communicating results to the requesting clinician can add lengthy delays which may impact care.

**Figure 1:**
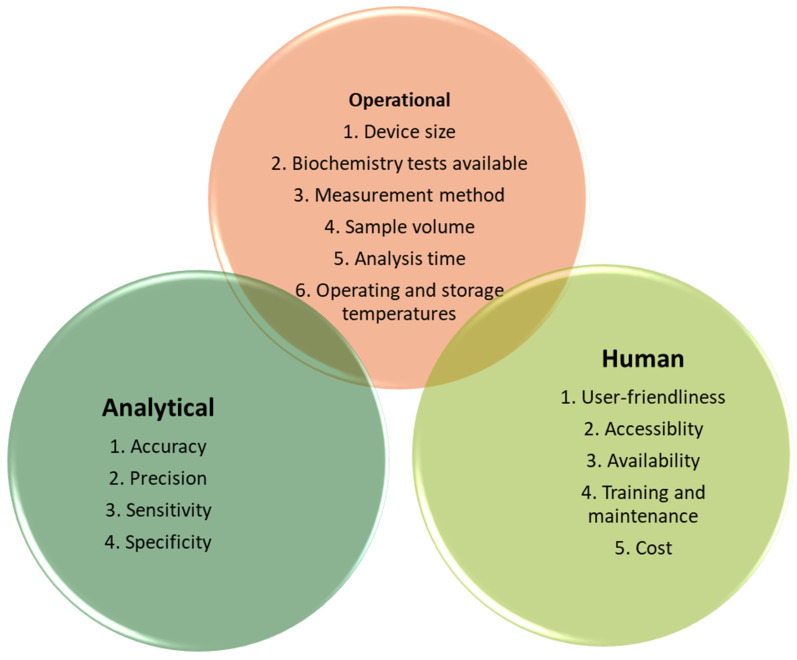
Illustrates the operational, analytical and human factors, which should be considered for each device in a given clinical context.

POCT is a rapid alternative to laboratory testing. There are a number of devices available, but due to variability in design (Figure 1) and sample analysis methodology for both POCT and laboratory assays, the accuracy and precision of POCT (intra-individual and inter-device) compared to laboratory testing is inconsistent.

Accuracy typically is expressed as absolute or percentage bias. The coefficient of variation (CV = standard deviation divided by mean) is a marker of precision. Analytical total error (ATE = bias + 1.96 × standard deviation) takes into account systematic bias (inaccuracy) and random bias (imprecision) to provide a more comprehensive assessment. Standardised reference ranges have been developed by various groups, including the Clinical Laboratory Improvement Amendments (CLIA), the Laboratory Working Group for the National Kidney Disease Education Program (NKDEP), and the Clinical & Laboratory Standards Institute (CLSI) [21,22,23]. A summary of ATE and acceptable percentage bias is described in Table 1.

The Statsensor (Nova Biomedical, Waltham, MA, USA) has been widely assessed for creatinine (and eGFR). It is small, portable, requires a small sample volume (1.2 μL), and is user-friendly. Imprecision (CV) has been reported as ranging from 3 to 13% over a wide range of creatinine concentrations [24,25,26,27,28,29,30,31]. However, it tends to be less accurate with older ages and greater creatinine concentrations. In vitro studies have suggested this is linked to high creatine and urea concentrations falsely elevating creatinine results [27]. A new model called the Nova Max Creatinine device has since been released by Nova Biomedical, with one study (*N* = 517) reporting 98.9% sensitivity and 85.0% specificity in detecting eGFR < 60 mL/min/1.73 m^2^) compared to laboratory reference standards [32].

The i-STAT Alinity (Abbott, Abbott Park, IL, USA) measures a wider array of biochemical markers, including creatinine and eGFR. It is also a light (660 g) hand-held device requiring 65 μL of blood and has a turnaround time of 2 min. The Epoc Blood Analysis System (Siemens Healthineers, Erlangan, Germany) is a slightly larger device but remains hand-held and portable. It requires a larger volume of blood (92 μL) and therefore would be less suitable for home self-testing.

Currin et al. compared both the i-STAT and Statsensor to the laboratory Jaffe method IDMS-traceable creatinine assays and iohexol-derived measured GFR in a rural South African population (*N* = 674). Both laboratory and POC tests overestimated Chronic Kidney Disease Epidemiology Collaboration (CKD-EPI) eGFR compared to measured GFR, though, the positive bias was less for i-STAT and Statsensor (8.4 vs. 19.9 vs. 28.6 mL/min/1.73 m^2^, respectively). However, the POCT devices showed wider imprecision (4.6% and 10.2% vs. 3.5%, respectively) [33]. Van der Heijden et al. compared both devices alongside the Epoc Blood Analysis System. I-Stat had the best accuracy (ATE = 6%), followed by Epoc, then Statsensor. All devices showed considerable variability around the creatinine reference standard, although Epoc performed best (95% limits of agreement −0.49 to 0.49 mg/dL).

A list of devices with a summary of operational and analytical characteristics is described in Table 2. These devices can be used across all age ranges, although there are fewer validation data in children, particularly with the handheld devices.

## 3. The Role of Point of Care Testing (POCT) in Kidney Disease

For kidney disease, POCT involves a blood test (for creatinine, eGFR, and potassium) and/or a urine test (UACR or urinary protein: creatinine ratio). POCT provide an opportunity to target early interventions in settings more convenient for service users, to improve health outcomes and patient quality of life [34].

The National Institute for Health Research’s Horizon Report in 2014 identified four areas where POCT would offer additional benefits to routine care: (1) screening for CKD by detection of elevated creatinine levels; (2) dose adjustment of prescribed medications in patients with renal impairment; (3) monitoring of CKD and (4) detection of AKI (including AKI on a background of CKD) [35].

### 3.1. Screening for CKD

Studies describing POCT CKD screening programs include FINISHED (First Nations Community Based Screening to Improve Kidney Health and Prevent Dialysis), Kidney Evaluation for You (KEY), and a pharmacist-led study in the United Arab Emirates [34,36,37]. FINISHED was a comprehensive screening, triage and treatment program for indigenous populations in rural and remote communities across Canada, to identify individuals with diabetes, hypertension and CKD. Out of 1700 people screened, 25.5% of adults (*N* = 343) were found to have CKD and 15% of children were identified to have early kidney disease (either through POC blood testing or urinalysis) [36,38]. This facilitated earlier treatment for CKD in a cohort with a higher prevalence of kidney disease but reduced access to healthcare, improved nephrology follow-up (8.4% versus 2.5% prior to screening) and was cost-effective with a gain of CAD 23,700 per quality-adjusted life year [39].

KEY was a community-based pilot study in diverse Australian regions (a rural mining town, regional centre, and metropolitan city) to screen high-risk individuals for conditions, including CKD. Similar to FINISHED, 20.5% of undiagnosed CKD cases were identified out of 402 participants enrolled in the study, using the i-STAT POC device for creatinine/eGFR or urine dipstick and subsequent UACR testing with the expectation of delaying future morbidity and mortality through early identification [34].

Pharmacy-led initiatives in community settings have also been performed in Canada and the United Arab Emirates. Donovan et al. piloted a study using the Statsensor device, identifying 10 CKD cases from 89 participants [40]. In the United Arab Emirates, patients with risk factors for CKD (diabetes mellitus, hypertension, or a positive family history of CKD) were targeted and POCT was performed on 400 participants at pharmacies using the PICCOLO (Abaxis) blood chemistry analyser (see Table 2), identifying undiagnosed CKD in 38.8% (*N* = 155) of participants [37].

Limitations included a lack of confirmatory testing for diagnosis (i.e., diagnosis was made with a single eGFR < 60 mL/min/1.73 m^2^), selection bias, and lack of data on sampling success and patient experience.

Furthermore, in three of the four studies, the POCT devices were operated by a healthcare professional, in most cases due to sample volume required ± device operation (the exception being Donovan et al. which used Statsensor), which precludes the option for home testing and potentially increases staff workload. This adds further workload and does not allow the option for home testing.

Despite these potential barriers, the studies discussed all highlight the potential benefits of using POCT in rural, remote, or urban community settings: (1) screening for the presence of CKD, (2) determining the severity and associated risk, and (3) initiating and optimising management through implementation of focused strategies to delay disease progression and manage risk factors, with the potential benefits of reduction in long-term morbidity and mortality.

Presently, screening for CKD using capillary POCT remains limited in the literature. This is likely related to the increased costs required to support this process and the potential increased workload that would result from greater identification. However, given the rapid advances in technology, the growing demand for more accessible care, and increasing awareness of the associated morbidity and premature mortality associated with CKD, it is likely that this is a field which will continue to grow as resources and financial support are redirected towards it.

### 3.2. Dose Adjustment of Prescribed Medications in Patients with Renal Impairment

**Figure 2:**
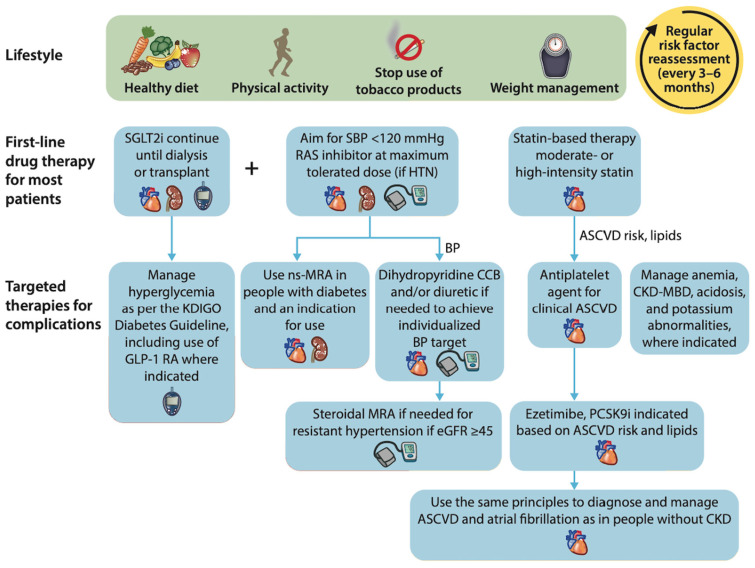
An illustration of the KDIGO treatment algorithm recommendations. This figure has been reproduced from the published KDIGO 2024 CKD Clinical Practice Guidelines for the Evaluation and Management of CKD [45]. Abbreviations: SGLT2i = sodium–glucose cotransporter 2 inhibitor; SBP = systolic blood pressure; RAS = renin-aldosterone-angiotensin system; HTN = hypertension; GLP-1 = glucagon-like peptide 1; MRA = mineralocorticoid receptor antagonist; CCB = calcium channel blocker; PCKS9i = proprotein convertase subtilisin/kexin type 9 gene; ASCVD = atherosclerotic cardiovascular disease; CKD-MBD = chronic kidney disease mineral bone disease; eGFR = estimated glomerular filtration rate.

**Figure 3:**
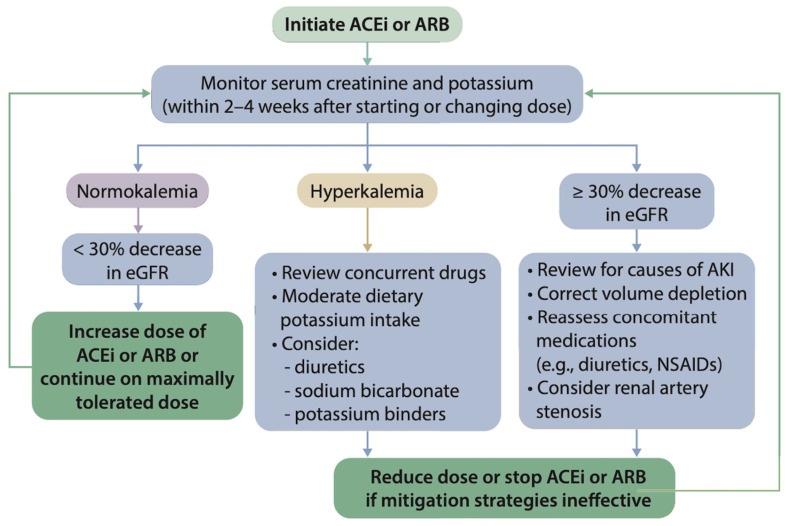
An illustration of the KDIGO treatment algorithm for the initiation and follow-up management of ACEi and ARB. This figure has been reproduced from the published KDIGO 2024 CKD Clinical Practice Guidelines for the Evaluation and Management of CKD [45]. Abbreviations: ACEi = Angiotensin-converting enzyme inhibitors; ARB = angiotensin receptor blocker; eGFR = estimated glomerular filtration rate.

The KDIGO 2023 Clinical Practice Guideline for the Evaluation and Management of CKD are for sodium–glucose co-transporter 2 inhibitors (SGLT2i) to be prescribed along with a maximum tolerated dose of angiotensin-converting enzyme inhibitors (ACEi) or angiotensin receptor blockers (ARB; see Figure 2) [12]. The combination of these medications for people with diabetic and non-diabetic proteinuric kidney disease has demonstrated a risk reduction in cardiovascular events, reduced proteinuria, and slowed the progression of kidney disease [41,42], adding up to 20 additional years before reaching ESRF for a middle-aged person optimised with an eGFR of 60 mL/min/1.73 m^2^ [43]. In addition, fineronone has recently been shown to also deliver similar outcomes in addition to ACEi or ARB [44]. However, these medication groups require renal function monitoring after initiation and incremental dose changes (see Figure 3), which provides a potential target for POCT to (1) guide rapid up-titration and (2) utilise patient-led home monitoring.

The screening programs discussed included a triage and treatment follow-up after identification [34,36,37,40,46]. Two were performed in a pharmacy setting, which has proven to be a popular location in community settings. This is logical, as pharmacies specialise in medication management and are often a trusted part of diverse local communities [47].

This is highlighted in a Dutch study, where 46 elderly patients on renally excreted medications had POCT, identifying over half with mild-to-moderate renal impairment, leading to medication adjustments and positive experiences reported by participants (4/5 using a Likert scale; 1 = bad, 5 = excellent) [48]. Ramos et al. used the NovaMax Statsensor device on 552 patients across 15 pharmacies in Spain. If dose adjustment or withdrawal was recommended, due to a decline in function or an eGFR below the recommended threshold, patients were referred to their physician, leading to modification of medications in 39 (7.1%) patients [49].

These community programs were able to successfully identify patients at risk on renally excreted medications in local community healthcare settings and established a strong foundation for future work to design and evaluate complex interventions using POCT to start renin-angiotensin-aldosterone inhibitors (RAASi) and monitor them through the up-titration process. However, additional POCT to monitor potassium concentrations is important, particularly for patients with advanced CKD and/or diabetes mellitus.

### 3.3. Monitoring of CKD

There is an opportunity for POCT in specific CKD cohorts requiring frequent surveillance, such as renal transplant recipients, patients on RAASi or diuretic therapies, or patients requiring potentially nephrotoxic treatments (e.g., intravenous injection of contrast dye). There is now an established and growing utility for POCT in each of these subgroups.

Kidney transplant recipients often have to travel greater distances than most patients and have to test more frequently, making this an ideal option for POCT. Key factors to consider in choosing a device are its accessibility, user-friendliness (in home settings), accuracy, and precision.

Murray et al. conducted a small observational feasibility study (*N* = 15) in stable kidney transplant recipients in the UK, using the Abbott i-STAT Alinity analyser at home for both creatinine and potassium concentrations compared to their standard laboratory testing [31]. The hand-held device was deemed easy to use by patients; however, the test success rate was 70% (42/60), with two-thirds of unsuccessful tests being due to device-related reasons. Within-patient bias for creatinine was 2.25 umol/L (95% CI: −12.13, 16.81 μmol/L); however, potassium was poorer at 0.66 mmol/L (95% CI: −0.147, 2.79 mmol/L) [31].

Lint et al. also reported that post-transplant patients (*N* = 30) were highly motivated and satisfied with home-testing using the Nova Biomedical Statsensor Xpress device, despite trust in the accuracy of creatinine being low, likely due to variation in subsequent measurements [50]. Lint et al. also performed a larger observational study with 138 kidney transplant recipients, comparing creatinine capillary POCT vs. venous POCT vs. IDMS-traceable enzymatic assays [30]. The coefficient of variation (CV) was 10.4% for capillary sampling and 5.2% for capillary sampling, which was above the authors’ defined allowable total error of 6.9% and the NKDEP’s desirable goal of 7.6%. These findings were similar to results published by Nataatmadja et al. in a study of 60 participants with CKD (20 with kidney transplants) who reported CV ranging from 5.8% to 11.3% [29]. In addition, there was no statistically significant difference between trends of capillary POCT samples compared to venous samples, suggesting that monitoring trends rather than detecting single sample changes in kidney function may be a more effective use of POCT testing [30].

Finger-prick capillary sampling using hand-held devices at home provides improved accessibility for patients, improving patient engagement and satisfaction and preserving venous access. Accuracy and precision in this cohort are vital, as there is a lower threshold for inaccurate results to cause anxiety, reduce patient and clinician confidence, and trigger further investigations. It is likely that a compromise between patient accessibility and user-friendliness versus accuracy and precision is required, and interpretation according to known limitations in test performance. Therefore, choosing the most appropriate device to meet these demands is key to a successful intervention.

## 4. Patient and Healthcare Professional Perspectives on POCT for Kidney Disease

To ensure the successful uptake and expansion of POCT in healthcare settings, confidence in the tests, through weighing up the benefits and limitations for both healthcare professionals and service users, is vital. Factors which will contribute to this decision include accuracy and precision, cost (to the service user and the healthcare system), accessibility (e.g., travel time, transport, and/or organising childcare), invasiveness of the test (e.g., finger-prick versus venepuncture), and previous experience.

Studies of patient and healthcare professional perspectives for kidney function assessment using POCT are limited; however, the perspectives from the wider use of non-creatinine POCT are valuable, as the themes regarding facilitators and barriers for implementation are likely to be similar.

In rural regions or areas where there are limited healthcare resources, one qualitative study based on 101 semi-structured interviews from patients, healthcare professionals, and policymakers in South Africa identified themes surrounding protracted turnaround times for ‘routine testing’, due to reduced accessibility and longer travel times (for patients and samples) [51].

However, participants from a mixed-methods service evaluation involving participants receiving home visits in the UK (*N* = 47) felt that POCT had benefited their care, with improved accessibility, reassurance with instant results, and reduced travel time as important factors [52]. This has been previously described in the Australian KEY screening program (*N* = 402), which reported improved satisfaction with POCT in 99% of participants, with increased convenience and better understanding of their condition (96% of participants) [34].

From a clinical perspective, alongside patient satisfaction, additional useful features for POCT include user-friendliness (linked with the amount of training required) and simple interpretation of results. Furthermore, the technical performance of devices, proven effect on clinical outcomes, and reliability of testing were all potential facilitators to the uptake of POCT [53].

Test reliability is key to providing user confidence, as both patients and healthcare professionals have expressed concerns regarding the risk of inaccurate results, which can lead to false reassurance, unnecessary anxiety, additional confirmatory testing, and extra workload [53,54].

Barriers to consider include increased costs (at least in the short term), legislative roadblocks, accessibility, extra workload, and access to maintenance and repairs for POCT equipment, particularly in rural settings [51].

## 5. Conclusions

POCT provides an accessible tool for achieving rapid results, enabling improved detection of CKD, monitoring of kidney function in high-risk groups, adjustment of renally excreted medications, and instant screening prior to interventions such as contrast-enhancing agent administration. It has been demonstrated to improve patient satisfaction, experience, and engagement in clinical services, and provides an attractive implementation tool for rural and remote regions where conventional testing is more challenging and health inequalities are compounded. POCT provides an exciting alternative to delivering healthcare within local communities, which may have the added benefit of reducing health inequalities that currently exist in nephrology.

Due to the range of devices and differences in their technical specifications, the choice of device will often be suited to the remit of the intervention in question, and these factors need to be considered by the care team to optimise effectiveness.

The scope for further expansion of kidney function testing includes optimisation of CKD medications and monitoring of renal function in patients on RAASi or diuretic therapies, initially targeting high-risk groups. Barriers included reduced accuracy and precision compared to laboratory testing, and increased short-term costs.

## Figures and Tables

**Table 1:** List of analytical total error (ATE) and coefficients of variation (CV) for creatinine and potassium, and estimated glomerular filtration rate (eGFR).

Analyte	Organisation	Limits of Acceptable Performance/Analytical Total Error (ATE)
Creatinine	CLIA, AAB	0.3 mg/dL (26.5 μmol/L) or 30%
	NKDEP	7.6% (desirable) 11.4% (minimum)
	BV	8.87%
	RCPA	10%
Potassium	CLIA, AAB	0.5 mmol/L
	BV	5.61%
	RCPA	≤4.0 mmol/L = ±0.2 mmol/L >4.0 mmol/L = 5%
eGFR	NKDEP	10%

Abbreviations: CLIA = Clinical Laboratory Improvements Amendment; AAB = American Association of Bioanalysts; NKDEP = National Kidney Disease Education Programme; BV = Spanish Society of Clinical Chemistry and Molecular Pathology (SEQC) Table of Desirable Quality Specifications based on Biological Variation; RCPA = The Royal College of Pathologists of Australasia and the Australasian Clinical Biochemist Association Quality Assurance Program.

**Table 2:** List of devices discussed with summary of operational and analytical product characteristics.

Device Name	Manufacturer *	Device Cost (£) **	Size	Testing Unit	Testing Unit Cost (£)	Creatinine Method	Sample Type (s)	Sample Volume	Analysis Time	Cr/eGFR Only	Storage Conditions (D = Device; T = Testing Unit)	Recommended Operating Temperature	Detection Range (umol/L)
Nova Statsenor	Nova Biomedical	1000–1500	Portable, Handheld	Strip	2.00–3.00	Amperometric/enzymatic	Whole blood	1.2 μL	30 s	Yes	D = Room temp. T = Refrigerated (2–8 °C)	15–40 °C	27–1056
Nova Max Creatinine	Nova Biomedical	2000–3000	Portable, Handheld	Strip	2.00–5.00	Amperometric/enzymatic	Whole blood	1.2 μL	30 s	Yes	D = Room Temp T = Refrigerated (2–8 °C)	15–40 °C 10–90% humidity	27–619
i-STAT Alinity	Abbott, Zoetis	5100–9000	Portable, Handheld	Cartridge	10.00–13.00	Amperometric/enzymatic	Whole blood, Plasma, Serum	65 μL	2 min	No–includes full renal profile	D = Room Temp T = Room Temp	16–30 °C	18–1768
Epoc Blood Analysis System	Siemens Healthineers	6600–9000	Portable, Handheld	Card	5.50–13.00	Amperometric/enzymatic	Whole blood, Plasma, Serum	90 μL (92 μL = Syringe)	40 s (165 s to calibrate)	No–includes full renal profile	D = Room Temp T = Room Temp	15–30 °C <80% humidity	27–1326
Piccolo Xpress	Abaxis	10,000–15,000	Table-top	Disc	10.00–20.00	Indicator Absorbance	Whole blood, Plasma, Serum	100 μL	12 min	No–includes full renal profile	D = Room Temp T = Refrigerated	15–32 °C 8–80% humidity	18–1768
ABL800 Flex	Radiometer	15,000–30,000	Table-top	Sensor Cassette	N/A ***	Amperometric/Enzymatic	Whole blood, Plasma, Serum	65–100 μL	1 min	Creatinine/Sodium/Potassium	D = 2–32 °C T = Room Temp	15–32 °C	10–2000

* Manufacturer locations are as follows: Nova Biomedical, Waltham, MA, USA; Abbott, Abbott Park, IL, USA; Zoetis, Parsippany, NJ, USA; Siemens Healthineers, Erlangan, Germany; Abaxis, Union City, CA, USA; Radiometer, Copenhagen, Denmark. ** Range of costs is indicative and may be subject to change depending on the specific supplier, promotions, and bulk purchasing agreements. For exact up-to-date pricing, the supplier should still be contacted directly. *** Unable to ascertain costs of sensor cassette.

## Data Availability

No new data were created or analyzed in this study. Data sharing is not applicable to this article.
